# Telemedicine: A promising future for inflammatory bowel disease management

**DOI:** 10.1016/j.amsu.2022.104684

**Published:** 2022-09-15

**Authors:** Faizan Fazal, Mohammad Ebad Ur Rehman, Omaima asif, Haris Mustafa, Usama tanveer, Tehseen haider, Abdul Rauf Khalid, Sajeel saeed, Jawad basit

**Affiliations:** Department of Medicine, Rawalpindi Medical University, Block E Satellite Town, Rawalpindi, Pakistan; Department of Pharmacology, Rawalpindi Medical University, Pakistan; Department of Medicine, Rawalpindi Medical University, Block E Satellite Town, Rawalpindi, Pakistan

**Keywords:** Health care, Disease burden, Developing countries

To the Editor,

Telemedicine is a term that refers to any activity of medical nature that involves communication despite being physically distinct [1]. In other words, it is unorthodox doctor-patient consultation, taking place virtually with patients that may be thousands of miles away from the doctor. Telemedicine means the adequate and appropriate delivery and provision of healthcare across distances [2].

Inflammatory bowel disease (IBD) is characterized by gastrointestinal illnesses that are chronic or remitting inflammatory diseases. IBD is further subdivided into two subtypes: ulcerative colitis (UC) and Crohn's disease (CD) [3]. Recent epidemiological studies have shown that cases of IBD are on a rise in developing countries such as Pakistan [4].

There are numerous difficulties in the timely diagnosis, treatment adherence, financial burden, regular follow ups, awareness, and misdiagnosis of inflammatory bowel disease [5]. All these problems can be linked to ineffective infrastructure, planning and strategies to manage patients with IBD. Studies in well-developed countries such as China show that patients with IBD bear massive financial burden due to the expensive nature of quality healthcare services, and this contributes to non-adherence to treatment [6]. Maintaining regular follow ups is another issue faced by patients with inflammatory bowel disease. A study in Brazil reported a significant reduction in irregular follow-ups during the COVID-19 era, which greatly hindered disease control [7]. Another issue is the lack of awareness regarding the etiology and treatment of IBD. Studies have demonstrated a significant association between low awareness, treatment non-adherence and anxiety in patients with IBD [8]. Anxiety and depression have further increased during the COVID-19 pandemic, particularly in elderly IBD patients [9].

All the major issues that are hindering the control of IBD can be addressed with telemedicine. Telemedicine interventions have shown a positive role in improving quality of life and reducing the number of required clinical visits in patients with IBD [10]. Telemedicine greatly benefits patients who do not have access to high-quality care due to financial or geographical barriers [11]. The results of telehealth interventions have shown to improve treatment adherence, disease activity, treatment surveillance, and knowledge related to the disease itself [12]. In addition to the patients, gastroenterologists have also demonstrated satisfaction with the incorporation of telemedicine in IBD management [13,14]. Telemedicine has also shown great promise in the management of pregnant females with IBD. An e-health portal is quite feasible for managing a pregnant IBD patient's reproductive and medication concerns during preconception and pregnancy [15].

In short, telemedicine is the most cost-effective, time-saving, and patient-friendly method to promoting patient compliance. Telemedicine can adequately resolve all problems that are encountered in the diagnosis and management of IBD patients. Investment into the necessary infrastructure to make telemedicine more accessible is essential at the government level. A reduction in the cost of telemedicine devices will be conducive to the widespread adoption of telemedicine in clinical practice. Healthcare workers and patients need to be trained to operate telemedicine devices optimally. Collaboration between all stakeholders is critical to ensure a smooth transition to telemedicine-based management of IBD patients.

## Ethical approval

Not applicable.

## Sources of funding

None.

## Author contributions

Faizan Fazal: Study conception, write-up, critical review and approval of the final version.

Mohammad Ebad Ur Rehman: Study conception, write-up, critical review and approval of the final version.

Omaima asif: Study conception, write-up, critical review and approval of the final version.

Haris Mustafa: Study conception, write-up, critical review and approval of the final version.

Usama Tanveer: Study conception, write-up, critical review and approval of the final version.

Tehseen Haider: Study conception, critical review and approval of the final version.

Abdul Rauf Khalid: Study conception, write-up, critical review and approval of the final version.

Sajeel saeed: Study conception, write-up, critical review and approval of the final version.

Jawad basit: Study conception, critical review and approval of the final version.

## Registration of research studies

1. Name of the registry: Not applicable
2. Unique Identifying number or registration ID: Not applicable
3. Hyperlink to your specific registration (must be publicly accessible and will be checked): Not applicable

## Guarantor

Faizan Fazal.

Mohammad Ebad Ur Rehman.

## Consent

Not applicable.

## Provenance and peer review

Not commissioned, externally peer reviewed.

## Declaration of competing interest

The authors have no conflicts of interest.

## References

[1] Wootton R. (2001 Sep 8). Telemedicine. BMJ.

[2] Craig J., Petterson V. (2005 Jan). Introduction to the practice of telemedicine. J. Telemed. Telecare.

[3] Nakase H., Uchino M., Shinzaki S., Matsuura M., Matsuoka K., Kobayashi T., Saruta M., Hirai F., Hata K., Hiraoka S., Esaki M. (2021 Jun). Evidence-based clinical practice guidelines for inflammatory bowel disease 2020. J. Gastroenterol..

[4] Park J., Cheon J.H. (2021 Feb 1). Incidence and prevalence of inflammatory bowel disease across Asia. Yonsei Med. J..

[5] Watermeyer G., Katsidzira L., Setshedi M., Devani S., Mudombi W., Kassianides C. (2022 Jun 30). Inflammatory bowel disease in sub-Saharan Africa: epidemiology, risk factors, and challenges in diagnosis. Lancet Gastroenterol. Hepatol..

[6] Yu Q., Zhu C., Feng S., Xu L., Hu S., Chen H., Chen H., Yao S., Wang X., Chen Y. (2021 Jan 5). Economic burden and health care access for patients with inflammatory bowel diseases in China: web-based survey study. J. Med. Internet Res..

[7] Feitosa M.R., Parra R.S., de Camargo H.P., da Costa Ferreira S., de Almeida Troncon L.E., da Rocha J.J., Féres O. (2021). COVID-19 quarantine measures are associated with negative social impacts and compromised follow-up care in patients with inflammatory bowel disease in Brazil. Ann. Gastroenterol..

[8] Sutton R.T., Wierstra K., Bal J., Ismond K.P., Dieleman L.A., Halloran B.P., Kroeker K.I., Fedorak R.N., Berga K.A., Huang V.W. (2021 Feb). Pregnancy-related beliefs and concerns of inflammatory bowel disease patients modified after accessing e-Health portal. J. Can. Assoc. Gastroenterol.

[9] Leung K., Gao Y., Hammami M.B. (2021 Oct 1). S788 prevalence of depression and anxiety among elderly veterans with inflammatory bowel disease during the COVID-19 pandemic. Official journal of the American College of Off. J. Am. Coll. Gastroenterol ACG.ogy| ACG.

[10] Pang L., Liu H., Liu Z., Tan J., Zhou L.Y., Qiu Y., Lin X., He J., Li X., Lin S., Ghosh S. (2022 Mar 24). Role of telemedicine in inflammatory bowel disease: systematic review and meta-analysis of randomized controlled trials. J. Med. Internet Res..

[11] Rowan C., Hirten R. (2022 Jul 1). The future of telemedicine and wearable technology in IBD. Curr. Opin. Gastroenterol..

[12] Davis S.P., Ross M.S., Adatorwovor R., Wei H. (2021 Feb). Telehealth and mobile health interventions in adults with inflammatory bowel disease: a mixed‐methods systematic review. Res. Nurs. Health.

[13] Guillo L., Bonnaud G., Nahon S., Caron B., Olympie A., Laurain A., Serrero M., Buisson A., Peyrin-Biroulet L. (2022 Apr 1). French experience with telemedicine in inflammatory bowel disease: a patients and physicians survey. Eur. J. Gastroenterol. Hepatol..

[14] Lahat A., Shatz Z. (2021 Feb). Telemedicine in clinical gastroenterology practice: what do patients prefer?. Ther. Adv. Gastroenterol..

[15] Sutton R.T., Wierstra K., Bal J., Ismond K.P., Dieleman L.A., Halloran B.P., Kroeker K.I., Fedorak R.N., Berga K.A., Huang V.W. (2021 Feb). Pregnancy-related beliefs and concerns of inflammatory bowel disease patients modified after accessing e-Health portal. J. Can. Assoc. Gastroenterol.

